# Marine fungus *Aspergillus* c1. sp metabolite activates the HSF1/PGC-1α axis, inducing a thermogenic program for treating obesity

**DOI:** 10.3389/fphar.2024.1320040

**Published:** 2024-01-25

**Authors:** Yong Rao, Rui Su, Chenyan Wu, Guanyu Yang, Renquan Fu, Junjie Wu, Jinqiang Liang, Jin Liu, Zhongping Jiang, Congjun Xu, Ling Huang

**Affiliations:** Key Laboratory of Tropical Biological Resources of Ministry of Education, School of Pharmaceutical Sciences, Hainan University, Haikou, China

**Keywords:** obesity, HSF1, PGC-1α, adipose browning, marine fungus

## Abstract

**Background and aims:** Obesity is one of the most prevalent diseases worldwide with less ideal approved agents in clinic. Activating the HSF1/PGC-1α axis in adipose tissues has been reported to induce thermogenesis in mice, which presents a promising therapeutic avenue for obesity treatment. The present study aimed to identified novel natural HSF1 activator and evaluated the therapeutic effects of the newly discovered compound on obesity-associated metabolic disorders and the molecular mechanisms of these effects.

**Methods:** Our previous reported HSF1/PGC-1α activator screening system was used to identify novel natural HSF1 activator. The PGC-1α luciferase activity, immunoblot, protein nuclear-translocation, immunofluorescence, chromatin immunoprecipitation assays were used to evaluate the activity of compound HN-001 in activating HSF1. The experiments of mitochondrial number measurement, TG assay and imaging, cellular metabolic assay, gene assays, and CRISPR/Cas 9 were applied for investigating the metabolic effect of HN-001 in C3H10-T1/2 adipocytes. The *in vivo* anti-obesity efficacies and beneficial metabolic effects of HN-001 were evaluated by performing body and fat mass quantification, plasma chemical analysis, GTT, ITT, cold tolerance test, thermogenesis analysis.

**Results:** HN-001 dose- and time-dependently activated HSF1 and induced HSF1 nuclear translocation, resulting in an enhancement in binding with the gene *Pgc-1α*. This improvement induced activation of adipose thermogenesis and enhancement of mitochondrial oxidation capacity, thus inhibiting adipocyte maturation. Deletion of HSF1 in adipocytes impaired mitochondrial oxidation and abolished the above beneficial metabolic effects of HN-001, including adipocyte browning induction, improvements in mitogenesis and oxidation capacity, and lipid-lowering ability. In mice, HN-001 treatment efficiently alleviated diet-induced obesity and metabolic disorders. These changes were associated with increased body temperature in mice and activation of the HSF1/PGC-1α axis in adipose tissues. UCP1 expression and mitochondrial biogenesis were increased in both white and brown adipose tissues of HN-001-treated mice.

**Conclusion:** These data indicate that HN-001 may have therapeutic potential for obesity-related metabolic diseases by increasing the capacity of energy expenditure in adipose tissues through a mechanism involving the HSF1/PGC-1α axis, which shed new light on the development of novel anti-obesity agents derived from marine sources.

## Introduction

Obesity is one of the most prevalent diseases worldwide and is considered a global burden and an epidemic. Obesity is a result of excessive lipid accumulation in adipose tissue, and fat build-up might contribute to an imbalance between calorie intake and energy expenditure. Adipocytes of different types have distinct opposite functions in regulating energy homeostasis (Sakers et al., 2022). White adipose tissue (WAT) is responsible for energy storage *via* the synthesis and accumulation of triglycerides, and is closely correlated with the development of obesity and insulin resistance. In contrast, brown adipose tissue (BAT) dissipates energy through the activation of uncoupling protein 1 (UCP1) found in mitochondria and burns fat through thermogenesis to defend against obesity (Chouchani et al., 2019). In this context, the induction of a thermogenic program in adipose tissue presents an attractive hotspot in the field of developing anti-obesity agents (Tam et al., 2012; Reilly and Saltiel, 2015).

Peroxisome proliferator-activated receptor γ coactivator-1α (PGC-1α) is a central regulator of mitochondrial biogenesis. It was reported to coordinate the expression of thermogenic genes including UCP1, and initiate the process of thermogenesis by interacting with UCP1 (Puigserver et al., 1998; Wu et al., 1999). Additionally, PGC-1α expression correlates with typical markers of beige-selective genes in human brown adipose tissue (Sharp et al., 2012). Knockout of PGC-1α impeded thermogenesis and exacerbated obesity in mice (Kleiner et al., 2012). In contrast, activation of PGC-1α by gene modulation or pharmacological interventions efficiently induced thermogenesis and counteracted obesity (Pettersson-Klein et al., 2018). Heat shock factor 1 (HSF1) is a transcription regulator, that controls PGC-1α expression by recognizing the heat shock element (HSE) in the promoter region (Ma et al., 2015). Our previous and other studies have revealed that activation of the HSF1/PGC-1α axis could enhance mitochondrial homeostasis and adaptive oxidation as well as induce a thermogenic program in adipose tissues, leading to ameliorations of metabolic disorders, including obesity and metabolic associated fatty liver disease (MAFLD) (Ma et al., 2015; Li et al., 2022a; Rao et al., 2022). Thus, activation of the HSF1/PGC-1α axis presents a promising strategy for developing potential anti-obesity agents. However, few natural specific HSF1/PGC-1α axis activators have been reported to date.

Marine natural products are an important source of compounds for innovative drugs, and 18 active natural products of marine origin or their derivatives have been approved by the National Medical Products Administration of the United States, the European Union and Japan as antitumor, antiviral, antibacterial, and anti-Alzheimer’s disease (AD) drugs, including Didemnin B, cephalosporin C, cytarabine, arabinosine, ziconotide, ericrine mesylate, bentuximab, GV-971, etc (Chun et al., 1986; Li et al., 2019). However, marine-derived compounds for MAFLD treatment have rarely been reported. The first aim of the present study was to identify novel natural HSF1/PGC-1α axis activators from a marine-derived compound library using the high-throughput HSEs/HSE^Del^-PGC-1α-luciferase screening system reported by us Li et al. (2022b). Second, we aimed to explore whether activation of HSF1 can induce a thermogenic program and be a practicable therapeutic strategy for obesity treatment. Based on the demonstration that the pharmacological stimulation of HSF1 could be used to treat obesity. Our third aim was to investigate the efficacy and potential mechanism by which pharmacological stimulation of HSF1 alleviated obesity.

## Materials and methods

### PGC-1α luciferase activity determination

The HSEs/HSEDel-PGC-1α-luciferase screening system was established as we previously reported (Li et al., 2022a). After transfection, the cells were treated with compound HN-001 (the purity of was over 95%) and PGC-1α luciferase activity was measured after 24 h treatment with dual luciferase system (Promega, E2910, China) by normalizing to protein levels.

### Immunoblot and immunofluorescence

For immunoblot, protein lysates from cells or tissues were prepared as previously described for immunoblotting using specific antibodies described as follows: HSF1 (Bioss, Cat#bs-3757R, China), pHSF1^Ser326^ (Abcam, Cat#ab76076, China), UCP1 (Abcam, Cat#ab234430, China), PGC-1α (Affinity, Cat#AF5395, China), CPT-1β (Abcam, Cat#ab134988, China), pLKB1^Ser431^ (Santa Cruz, Cat#sc-271924, China), pAMPKα^Thr172^ (Affinity, Cat#AF3423, China), FASN (Affinity, Cat#DF6106, China), ACC (Affinity, Cat#AF6421, China). For immunohistochemistry analysis, the paraformaldehyde-embedded adipose tissues were sliced (4 μm thick) with a rotary microtome (Leica, Germany). The sections were deparaffinized and hydrated. Heat-mediated antigen was retrieved with 10 mM citrate buffer pH 6.0 (Thermo Scientific, Cat#005000, Guangzhou, China). Endogenous peroxide was inhibited by incubating with a freshly prepared 3% H_2_O_2_ solution in MeOH. Non-specific antigens were blocked by incubating sections with IHC blocking buffer (Thermo Scientific, Cat#00-4953-54, Guangzhou, China) for 1 h. Then the adipose sections were stained with p-HSF1^Ser326^ and UCP1 antibody, followed by a goat anti-rabbit IgG HRP conjugate (Thermo Scientific, Cat#65-6120, Guangzhou, China). Color was developed after an incubation with 3, 3′-diaminobenzidine (DAB) substrate (ThermoFisher Scientific, Cat#SK34065, Guangzhou, China), followed by hematoxylin counterstaining and mounting (Vector laboratories, Cat#H-5000, RRID: AB_2336786, China). The stained sections were photographed as described above. The positive (stained) area for each marker was imaged with a minimum of 10 random liver sections per sample. Images presented in the figures showed the animals with a median value for each group.

### Chromatin immunoprecipitation (ChIP) assay

ChIP was performed using the ChIP kit (ThermoFisher Scientific, Cat#26157, China) following the manufacturer’s instructions as we previously reported (Rao et al., 2022). Antibodies to HSF1 (Abcam, Cat#ab26757, China) or normal rabbit IgG (Cell Signaling technology, Cat#6990, China) was used as a negative control as we previously reported. The immunoprecipitated DNA was amplified by real-time PCR using primers specific for the mouse *Pgc-1α* promoter region. The relative HSF1 at the *Pgc-1α* promoter was determined. The average enrichment levels of HSF1 in control cells or mice were set as 1, and the relative folds were calculated.

### Mitochondrial number measurement

Mitochondrial DNA (mtDNA) copy numbers were quantified by PCR. Briefly, DNA was extracted from cells or inguinal WATs or BAT using a DNeasy Blood and Tissue kit. The copy numbers of nuclear DNA (nDNA) and mtDNA were assessed by PCR using primers targeting the cytochrome C gene (for mtDNA) and 18S rRNA (for nDNA) as we previously reported (Rao et al., 2017). Actin was selected as the loading control. To visualize mitochondria, cells were stained with Hochest 33342 (for nucleus, 1:1000) and mitochondria probe Mito-tracker (Thermo Scientific, 1:5000) at 37°C for 10 min. The images were then captured at the wavelength of Ex 350 nm/Em 405 nm and Ex 595 nm/Em 647 nm.

### Triglyceride (TG) assay and imaging

The adipocytes were lysed and centrifuged at 3000 *g* for 10 min at 4°C. The supernatants were isolated and subjected to quantification of total TG using TG assay kit (Roche, Cat#20767107322, China) according to the manufacturers’ protocol. For Nile red staining, after treatment, cells were stained with Hochest 33342 (for nucleus, 1:1000) and lipid dye (Nile red, 1:2000) at 37°C for 10 min. The images were then captured at the wavelength of Ex 350 nm/Em 405 nm and Ex 515 nm/Em 555 nm.

### Cellular metabolic assay

After 24 h treatment, the extracellular acidification rate (ECAR) and oxygen consumption rate (OCR) were determined by a Seahorse Bioscience XFpro Extracellular Flux Analyzer (Seahorse Bioscience) with 2 μM oligomycin, 1.5 μM carbonyl cyanide-4-(trifluoromethoxy) phenylhydrazone (FCCP) and 1 μM antimycin A/rotenone injected at regular intervals. The OCR was determined for 114 min at 6-min intervals by normalizing to protein levels in each well.

### Cas9/CRISPR

The specific mouse CMV-mKate 2-Cas 9, tracrRNA and HSF1 crRNA plasmid DNA were purchased from Horizon (Shanghai, China). C3H10-T1/2 cells were transfected with Cas 9 plasmid (3 μg/2×10^5^ cells) for 24 h, and then added with the mixture of HSF1 crRNA and tracrRNA for another 24 h using the DharmaFECT Duo Transfection Reagent (Horizon, Cat#T-2010–02, Shanghai, China). The cells were then subjected to adipocyte differentiation for the following examinations.

### Gene assay

Total RNA from cells or adipose was isolated using the TRIzol method (Invitrogen, Cat# 15596018, China) and the results were analysed using the 2^−ΔΔCT^ method on an ABI StepOne Plus real-time PCR system as in our previous report (Li et al., 2022b). Actin was used as a loading control. The average gene levels in control cells or mice were set as 1, and the relative folds were calculated. The primer information was listed as we previously reported.

### Animal study

All animal care and experimental procedures were carried out in accordance with the Guide for the Care and Use of Laboratory Animals of the National Institutes of Health. The protocols were approved by the Hainan University Committee on Animal Ethics for the Use of Laboratory Animals and were conducted in accordance with the Animal Welfare Legislation of China. Every effort was made to minimize the use of the animals and their discomfort. Eight-week-old male C57BL/6J mice were purchased from YanCheng BioTech Co., Ltd. (Guangzhou, China) and housed under specific pathogen-free conditions and reared in line with standardized methods at 22°C ± 1°C under a 12-h light/dark cycle with free access to food and water. After 1 week of acclimatization to the environment of this study, the mice were fed either a regular chow (CH) or HFC diet (Research Diet, United States). After 12 weeks of feeding, the CH or HFC mice were randomly divided into two or three subgroups and treated with saline or HN-001 (*i. p.*) each other day for 8 weeks. After treatment, body weight and food intake were monitored every other day, each group included 10 mice. At the end of the study, the mice were fasted for 6 h (08:00–14:00) and anaesthetized by an *i. p.* injection of ketamine/xylazine. After the mice were fully anaesthetized, their eyeballs were removed to collect blood samples into a tube containing 1 mM EDTA for the measurement of relevant plasma parameters. After collecting blood samples, the anaesthetized mice were sacrificed by cervical decapitation and the tissues of interest were weighed, freeze-clamped or fixed in 4% formaldehyde solution.

### Cold tolerance test

For cold tolerance test, the obese mice (after 12 weeks HFC diet feeding) were subjected to a cold room (4°C) or 22°C (referred to as “room temperature”) for 4 h with free access to food and water. The rectal temperature was measured at each indicated time point.

### GTT and ITT assay

A glucose tolerance test (GTT) was performed after 3 weeks of treatment with HN-001. Mice were fasted for a period of 6 h (08:00–14:00) and then injected with glucose (2 g/kg, *i. p*.). Insulin tolerance tests (ITT, insulin load 0.6 U per mouse, *i. p.*) were performed, after 6 h fasting (08:00–14:00), after 5 weeks of treatment with HN-001. The glucose concentrations were measured by venous puncture at 0, 15, 30, 45, 60, 90, and 120 min after treatment.

### Feces TG, NEFA, and cholesterol determination

The weighted fresh mouse feces dissolved in PBS (pH7.4) and vortexed at room temperature for 1 min, the solution was centrifuged at 3000 *g* for 15 min. The supernatants were collected for feces TG, non-esterification fatty acid (NEFA), and cholesterol (CHO) determination following the manufacturer’s instructions.

### Histological analysis

The adipose tissues were fixed in 4% formaldehyde solution and embedded in paraffin after dehydration in a graded ethanol series (70%–100%). Embedded samples were sectioned (4 μm thick) with a rotary microtome and stained with hematoxylin and eosin (H&E) for microscopic examination. Sections were viewed with a light microscope (Olympus) and photographed at ×200 magnification. The section of immunohistochemistry image was first labelled by an identification number code without the information of the grouping. The sizes of adipocytes of each slide were calculated with IMAGE J software. Quantification analysis was performed in six randomly selected fields per sample in a blinded manner.

### Data and statistical analysis

For cellular experiments, data were obtained from *n* = 3 independent biological experiments. For animal study, data were obtained from at least 8 independent mice in each group and the specific numbers were presented in Figure legends. The statistical analysis with parametric variables was performed using the original experiment data (non-normalized data) unless otherwise specific stated in the Figure legends. For the PCR assays, the average level of genes in control cells or control mice were set as 1, and the relative folds were calculated by comparing with the control group. Data included in data analysis and presentation are expressed as the mean ± SEM. Differences between two groups were analyzed by Student’s t-test using GraphPad Prism (GraphPad Software Inc., California, United States, RRID:SCR_002798). Statistical analysis for multiple groups was performed by one-way ANOVA followed by Tukey’s HSD *post hoc* tests. A *p*-value of ≤0.05 was considered statistically significant.

## Results

### Marine fungus *Aspergillus* sp. c1 metabolite HN-001 is a novel natural HSF1 indirect activator

**FIGURE 1:**
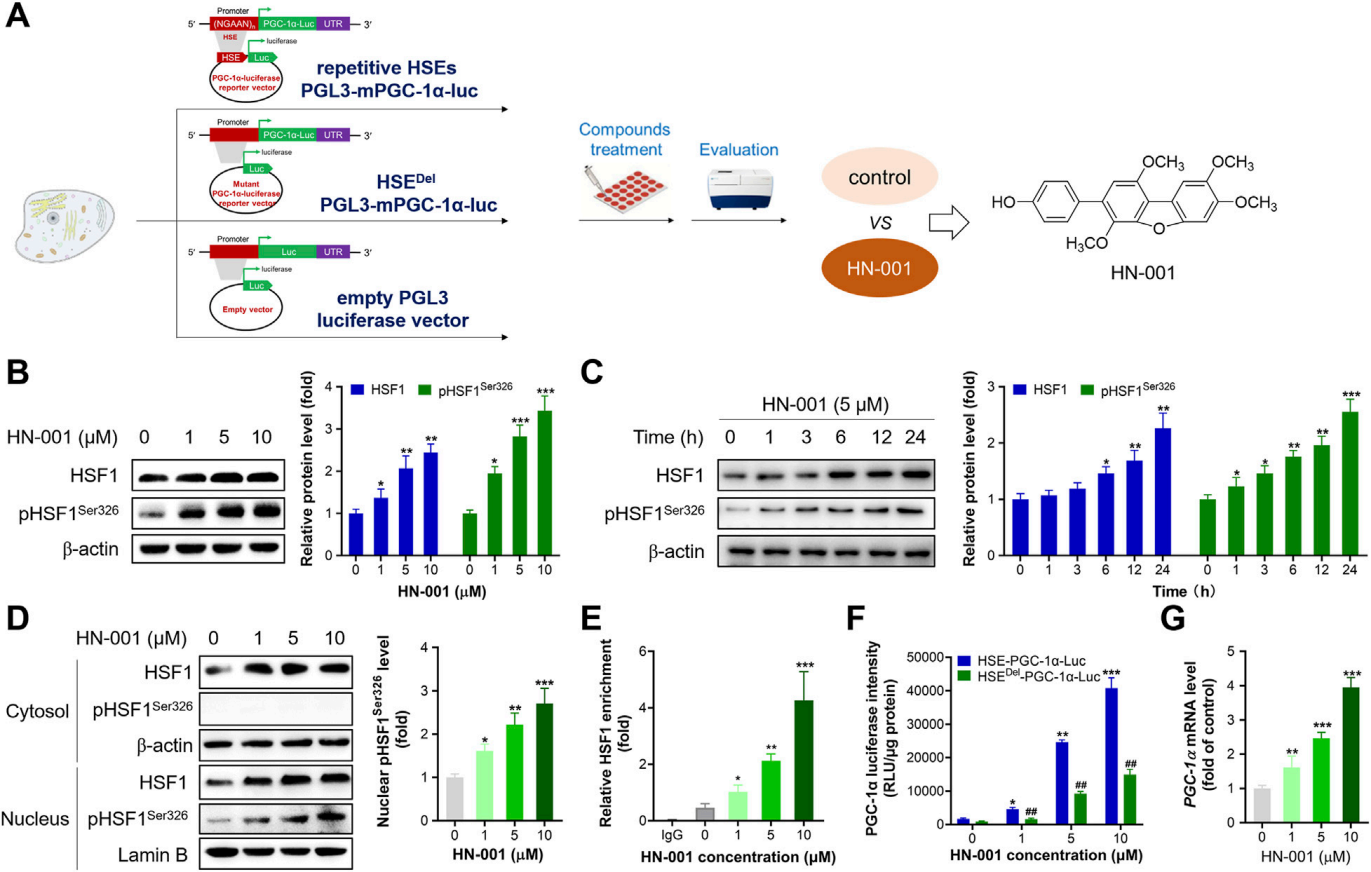
HN-001 activates the HSF1/PGC-1α axis in C3H10-T1/2 cells. **(A)** Schematic diagram of identification of HN-001 as a novel HSF1/PGC-1α axis activator. **(B, C)** Dose- and time-dependent effects on HSF1 and its phosphorylated level (Ser326) and quantification. **(D)** Examination of HSF1 and its phosphorylation level in the cytosol and nucleus, and quantification. **(E)** Enrichment of HSF1 in the promoter region of *Pgc-1α* DNA. N = 3 independent biological experiments. **(F)** Examination of PGC-1α luciferase activity in cells transfected with the HSE-PGC-1α-Luc or HSE^Del^-PGC-1α-Luc plasmid. **(G)** mRNA levels of *Hsf1* in adipocytes after 24 h treatment of HN-001. *p < 0.05, **p < 0.01, ***p < 0.001, compared with control cells. HN-001 inhibits adipocyte maturation and improves the thermogenic program.

By using the high-throughput HSF1/PGC-1α axis activator screening system reported by us, we identified a novel natural compound, HN-001 (chemical structure is listed in Figure 1A), as a novel HSF1 activator. Treating HN-001 in adipocytes dose- and time-dependently increased the protein levels of HSF1 as well as its phosphorylation (Ser326, active format) (Figures 1B, C). Examination of the level of p-HSF1^Ser326^ in the cytosol and nucleus revealed that HN-001 treatment dose-dependently increased the level of p-HSF1^Ser326^ in the nucleus, while p-HSF1^Ser326^ was not detected in the cytosol, indicating that HN-001 treatment facilitated HSF1 nuclear translocation and phosphorylation (Figure 1D). Consistent with the activation of HSF1, HN-001 treatment increased the enrichment of HSF1 protein in the promoter region of the *Pgc-1α* gene (Figure 1E). This leads to an increment in PGC-1α transcription levels as indicated by luciferase and qRT-PCR assays (Figures 1F,G). Notably, HN-001 treatment dose-dependently increased the luciferase activity of PGC-1α, while this increase was greatly weakened in the mutant HSE^Del^-PGC-1α-luciferase-transfected cells, as shown by an approximately 3-fold decrease in luciferase activity. Meanwhile, the luciferase activities of HN-001-treated cells transfected with HSE^Del^-PGC-1α-luciferase were greater than those of HSE^Del^-PGC-1α-luciferase control cells. Moreover, HN-001 treatment also increased the transcription level of *Hsf1* (Supplementary Figure S1), indicating that HN-001 may not be a specific HSF1/PGC-1α axis activator. What’s more, HN-001 treatment marginally displayed toxicity against adipocytes as indicated by the value of half maximal inhibitory concentration (IC_50_) over 100 μM (Supplementary Figure S2).

**FIGURE 2:**
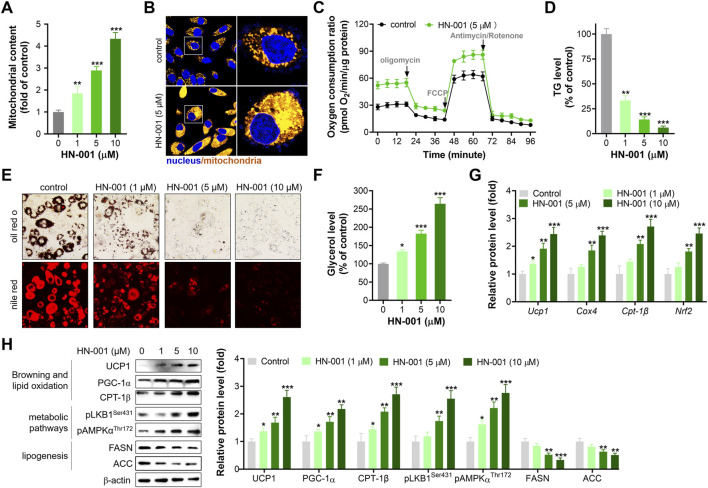
HN-001 enhances mitochondrial oxidation and inhibits adipocyte maturation alongside browning induction. **(A)** Mitochondrial copy numbers as indicated by mtDNA/nDNA. **(B)** Imaging mitochondria in adipocytes by using the probe Mito-Tracker^®^ Green FM. Scale bar, 50 μm. **(C)** Oxygen consumption ratio determination after 24 h treatment. **(D)** Cellular TG level assay. **(E)** Oil red O and Nile red staining. Scale bar, 100 μm. **(F)** Glycerol level determination. **(G)** mRNA levels of metabolism-related genes. **(H)** Expression levels of HSF1/PGC-1α axis- and metabolism-related proteins, and quantification. N = 3 independent biological experiments. *p < 0.05, **p < 0.01, ***p < 0.001, compared with control cells.

PGC-1α is a pivotal mitogenesis regulator, and activation of the HSF1/PGC-1α axis could induce thermogenesis by increasing mitochondrial copy numbers and oxidation capacity (Ma et al., 2015). As expected, treating adipocytes with HN-001 dose-dependently increased mitochondrial copy numbers (Figures 2A, B). Additionally, HN-001 addition enhanced mitochondrial oxidation capacity, including basal oxidation capacity, maximum oxidation capacity and ATP production leaked oxidation (Figure 2C). These improvements led to a reduction in cellular TG levels (Figures 2D, E) and an increase in glycerol levels (Figure 2F). Gene expression analysis revealed that HN-001 treatment activated a network of genes involved in mitogenesis (*Pgc-1α* and *Nrf2*) and mitochondrial oxidation (carnitine palmitoyltransferase-1β (*Cpt-1β*) and *cytochrome c oxidase subunit 4 (Cox4)*). Intriguingly, we noted that HN-001 treatment increased the transcription level of a thermogenesis marker (*Ucp1*) (Figure 2G). Meanwhile, immunoblot analysis demonstrated that treating HN-001 in adipocytes activated the metabolic pathway liver kinase B1 (LKB1)-AMP dependent protein kinase (AMPK), as indicated by increases in the phosphorylation levels of LKB1 and AMPK and increased protein levels of mitogenesis and mitochondrial oxidation markers, including PGC-1α and CPT-1β (Figure 2H). Notably, examination of the thermogenesis marker UCP1 in adipocytes further confirmed the induction effect of HN-001 on UCP1 levels. These enhancements led to a reversal in the protein levels of lipogenesis markers, including fatty acid synthase (FASN) and acetyl CoA carboxylase (ACC), which coincided with the reduction in TG levels.

### Deletion of Hsf1 impaired the thermogenic ability of HN-001

**FIGURE 3:**
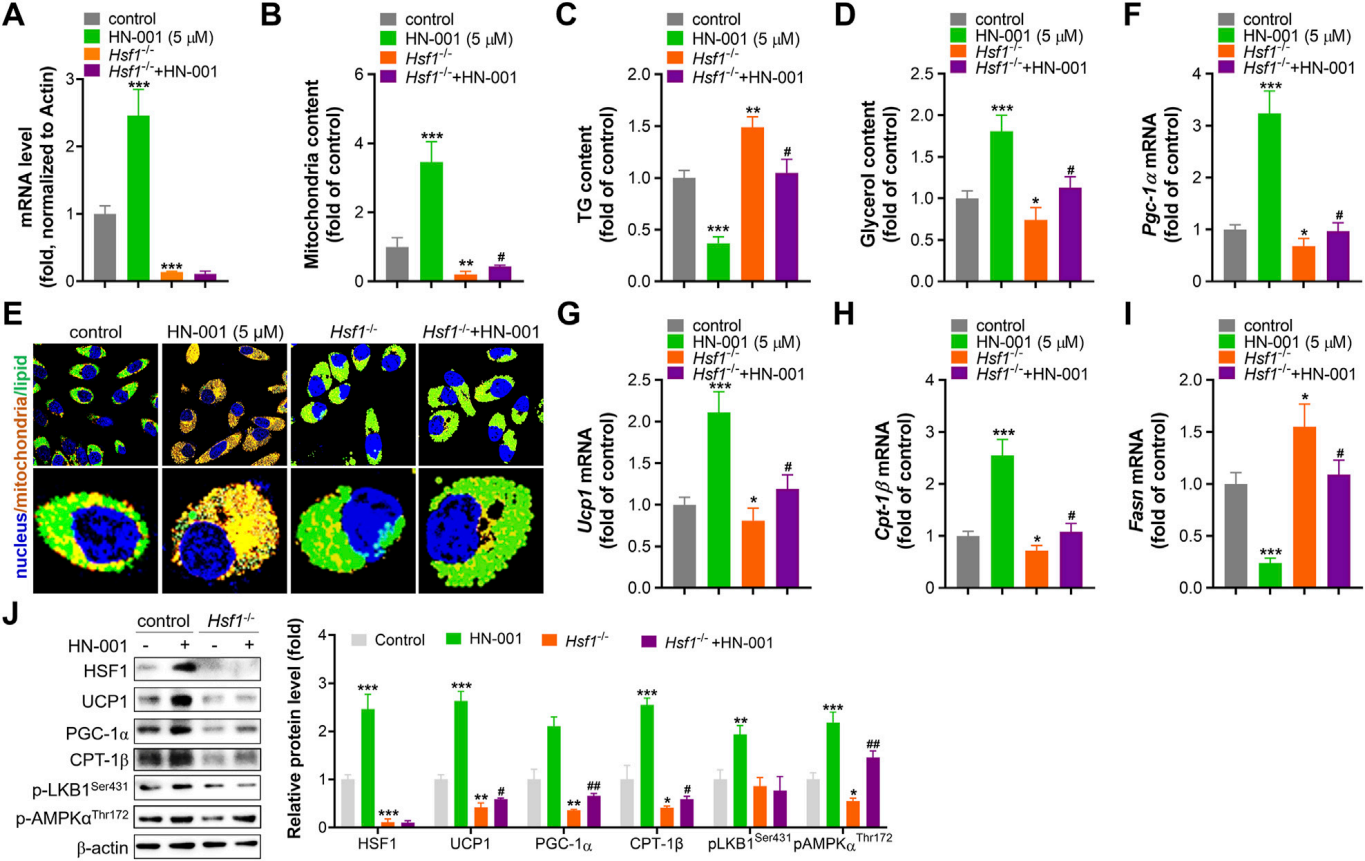
HSF1 deficiency abolishes the beneficial metabolic effects of HN-001. **(A)** mRNA level of HSF1 after 24 h of transfection. **(B)** Mitochondrial copy numbers after 9 days of adipocyte differentiation. **(C)** Cellular TG quantification after 9 days of adipocyte differentiation. **(D)** Cellular glycerol quantification after 9 days of adipocyte differentiation. **(E)** Images of lipids and mitochondria in adipocytes after 3 days of differentiation. Scale bar, 50 μm. **(F–I)** mRNA levels of *Pgc-1α*, *Ucp1*, *Cpt-1β*, and *Fasn* after 3 days of adipocyte differentiation. **(J)** Expression levels of HSF1/PGC-1α axis- and metabolism-related proteins, and quantification. N = 3 independent biological experiments. *p < 0.05, **p < 0.01, ***p < 0.001, compared with control cells; ^#^ *p* < 0.05, compared with *Hsf1* ^−/−^ control cells.

To further confirm the thermogenesis induction effect and HSF1 activation mediated by HN-001 treatment, we deleted HSF1 level in adipocytes by transfecting HSF1 CRIPSR plasmid as we previously reported. HSF1 deficiency impaired mitogenesis and increased lipogenesis, as indicated by decreased mitochondrial copy numbers and increased cellular TG level (Figures 3A–D). Also, HSF1 deletion effectively abolished the induction effect of HN-001 on *Hsf1* transcription level, weakened the mitogenesis and TG-decreasing effects of HN-001. Imaging cellular TG and mitochondria by its probes also confirmed the abrogating effect in increasing mitochondrial contents and decreasing TG level (Figure 3E). Consistent, gene analysis demonstrated that HSF1 deficiency downregulated the transcription level of *Pgc-1α*, *Ucp1*, *Cpt-1β* but increased the expression of lipogenesis marker *Fasn* (Figures 3F–I). And the induction or suppression effect of HN-001 on these genes were diminished. Analyzing the thermogenic markers and metabolic pathway also confirmed that deletion of HSF1 diminished the metabolic effects of HN-001 in adipocytes (Figure 3J). These data suggest that HN-001 inhibits adipocyte maturation by inducing thermogenesis, which was associated with HSF1/PGC-1α axis.

### HN-001 alleviates obesity and metabolic disorders in diet-induced obese (DIO) mice

**FIGURE 4:**
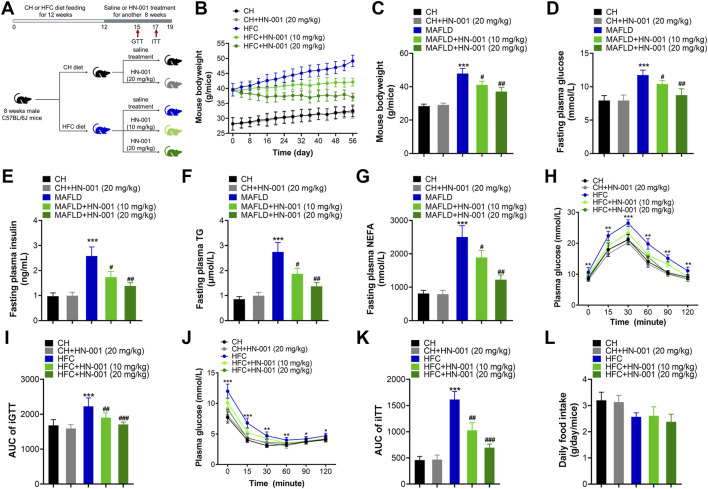
HN-001 ameliorates obesity and metabolic disorders in DIO mice. Eight-week-old male C57BL/6J mice were fed a chow diet (CH) or HFC diet for 12 weeks and then treated with saline or HN-001 (10 or 20 mg/kg, *i.p.* each other day for 8 weeks. **(A)** Schematic diagram of HN-001 treatment in mice. **(B, C)** Mouse body weight curve and the termination point body weight. **(D–G)** Fasting plasma glucose, insulin, TG, and FFA levels. **(H, I)** GTT and its AUC at week 3 of HN-001 treatment. **(J, K)** ITT and its AUC at week 5 of HN-001 treatment. (**L**) Mouse daily food intake. N = 10 mice/group. *p < 0.05, **p < 0.01, ***p < 0.001, compared with chow diet-fed mice; ^#^ *p* < 0.05, ^##^ *p* < 0.01, ^###^ *p* < 0.001, compared with HFC control mice.

The metabolic effects of HN-001 were evaluated by administration of the compound *via* intraperitoneal injection to obese mice induced by a high-fat and high-cholesterol (HFC) diet for 8 weeks (Figure 4A). As expected, HFC-fed mice gained significantly more body weight, but this was progressively delayed after 8 weeks of administration of HN-001, as indicated by approximately 10.6% and 20.1% body weight reductions (Figures 4B, C). Meanwhile, HN-001 treatment also significantly reduced the HFC diet feeding-induced increases in plasma levels of glucose, insulin, TG, and NEFA (Figures 4D–G). Mice treated with HN-001 had better tolerance to glucose load, which was particularly prominent at almost all time points of an *i.p.* GTT (Figures 4H, I). Additionally, HN-001 improved insulin sensitivity, as indicated by the ITT assay (Figures 4J, K). In addition, weight loss was not involved in any changes in daily energy intake (Figure 4L). These data showed that HN-001 reduced body weight and improved disordered metabolic profiles.

### HN-001 induces a thermogenic program in DIO mice

**FIGURE 5:**
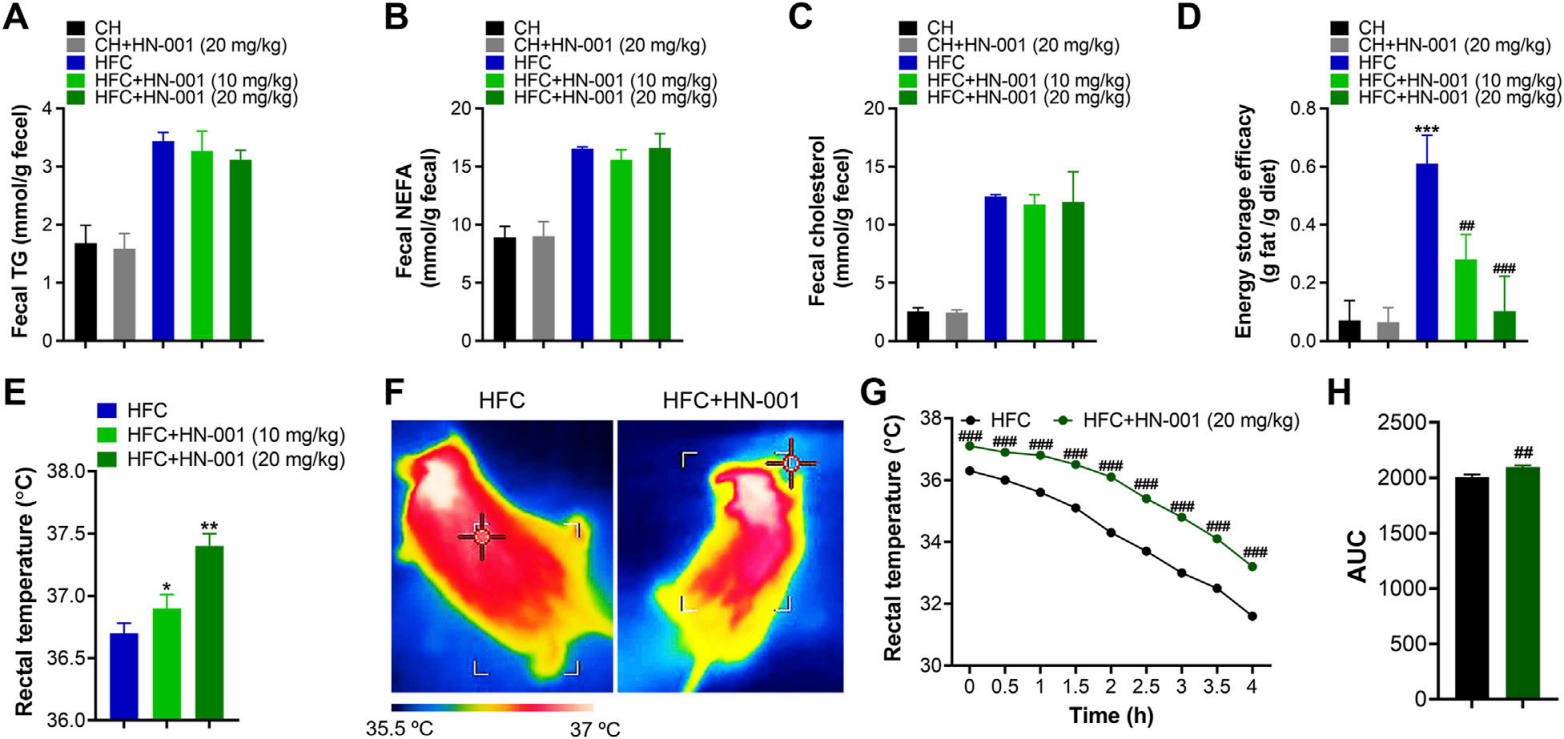
HN-001 induces a thermogenic program in mice. **(A–C)** Determination of fecal TG, NEFA, and cholesterol after 8 weeks of treatment. **(D)** Determination of energy storage efficacy. **(E)** Evaluation of rectal temperature. **(F)** Representative infrared thermal images. **(G, H)** Body scapula temperature during cold exposure. The HFC mice or HN-001-treated HFC mice (after 8 weeks of treatment) were exposed to a cold room, and the scapula temperatures were measured within 4 h. N = 8–10 mice per group. *p < 0.05, **p < 0.01, ***p < 0.001, compared with chow diet-fed mice; ^#^ *p* < 0.05, ^##^ *p* < 0.01, ^###^ *p* < 0.001, compared with HFC control mice. HN-001 induces a thermogenic program in white adipose tissue.

Restricting energy absorption and/or promoting energy consumption is supposed to be a common way to combat obesity in the clinic. Although HN-001 did not alter daily appetite in mice, we were eager to examine the actions of HN-001 on energy absorption. Interestingly, compared with that of vehicle-treated control mice, HN-001 treatment marginally affected energy absorption, as indicated by no change in the levels of fecal TG, NEFA, and CHO (Figures 5A–C). Meanwhile, we examined the energy storage efficacy in mice by normalizing the increased body weight to daily food intake and found that HFC diet-induced obese mice displayed a higher energy storage efficacy, while HN-001 treatment dose-dependently decreased energy storage efficacy (Figure 5D), indicating that HN-001 enhanced energy expenditure. In light of our observed data in cells, we then examined body temperature using the FLECS system and found that mice treated with HN-001 increased scapula temperature (Figures 5E, F). We further performed a cold tolerance test to gauge adaptive thermogenesis, which is another major component of energy expenditure. During 4 h of exposure to cold, the body temperatures of the control obese mice dropped significantly, while those of the HN-001-treated obese mice decreased slightly, indicating that HN-001 improved thermogenesis in mice (Figures 5G, H).

**FIGURE 6:**
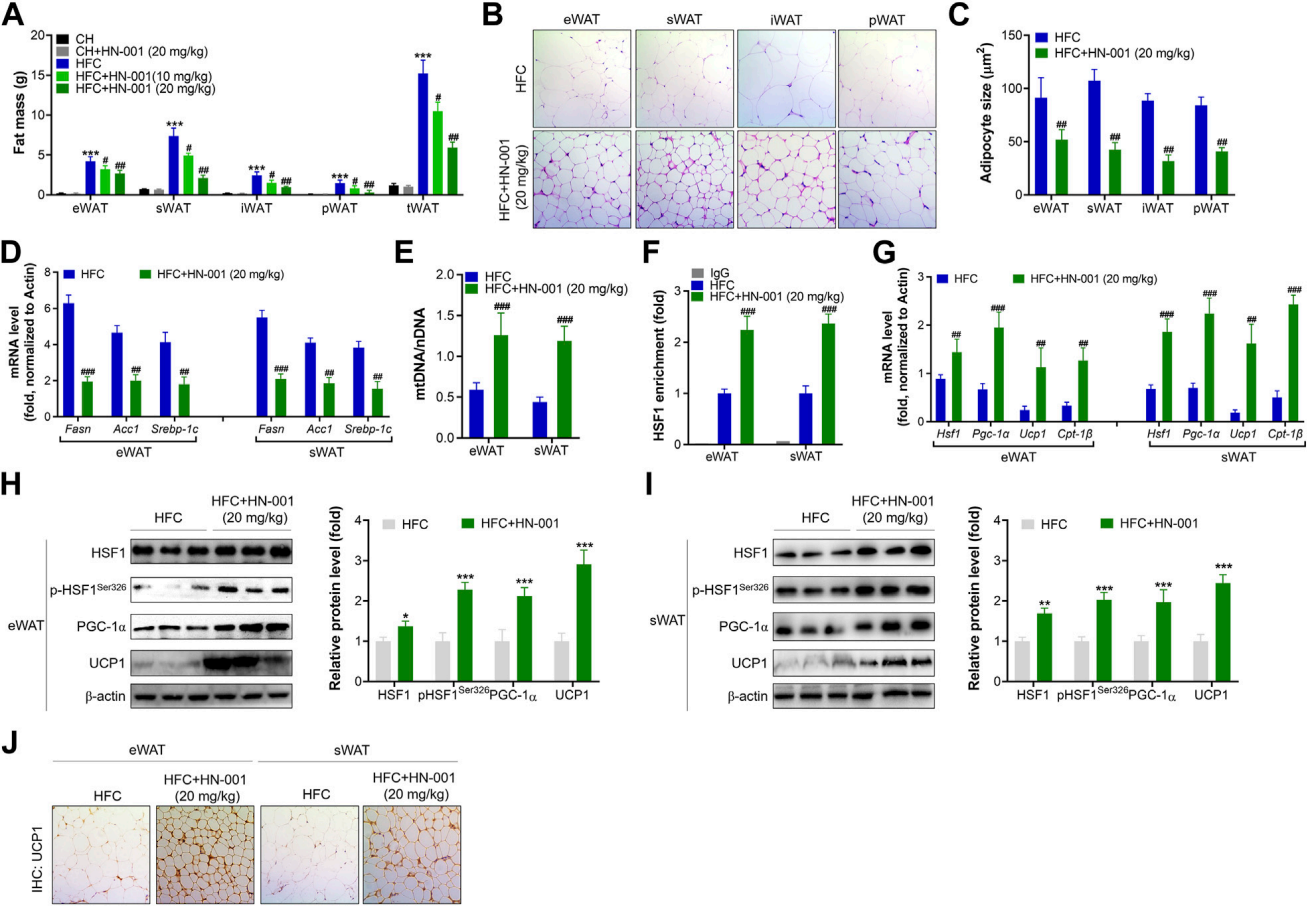
HN-001 induces a thermogenic program in WAT of mice. **(A)** Fat mass content. **(B)** H&E staining of WATs. Scale bar, 200 μm. **(C)** Adipocyte size measurement. **(D)** Expression of lipogenesis-related genes in eWAT and sWAT. **(E)** Mitochondrial content. **(F)** Enrichment of HSF1 in the promoter of the *Pgc-1α gene*. **(G)** mRNA levels of metabolic and browning regulatory genes. **(H, I)** Protein levels of HSF1/PGC-1α and browning markers in eWAT and sWAT, and quantification. **(J)** Immunohistochemistry analysis of UCP1 in eWAT and sWAT. Scale bar, 200 μm. N = 10 mice/group. *p < 0.05, **p < 0.01, ***p < 0.001, compared with chow diet-fed mice; ^#^ *p* < 0.05, ^##^ *p* < 0.01, ^###^ *p* < 0.001, compared with HFC control mice. HN-001 enhances the thermogenic capacity of BAT.

Consistent with the reduction in body weight, obese mice treated with HN-001 displayed a marked reduction in fat mass, including subcutaneous WAT (sWAT), epididymal WAT (eWAT), inguinal WAT (iWAT), and perirenal WAT (pWAT) (Figure 6A). In addition, the size of adipocytes in WATs of HN-001-treated obese mice was decreased (Figures 6B, C). This was associated with the suppression of the expression levels of lipogenic markers in eWAT and sWAT, including *Fasn*, *Acc1*, and *Srebp-1c* (Figure 6D).

HN-001 treatment increased mitochondrial copy numbers in eWAT and sWAT (Figure 6E). These results were correlated with improved binding of HSF1 to the *Pgc-1α* gene (Figure 6F). Gene analysis revealed that HN-001 treatment induced activation of the gene network involved in mitochondrial biogenesis and fatty acid oxidation as well as activation of browning markers in eWAT and sWAT (Figure 6G). Immunoblot analysis revealed that HN-001 treatment activated the HSF1/PGC-1α axis and increased the levels of the browning marker UCP1 in eWAT and sWAT (Figures 6H, I). Additionally, immunohistochemistry analysis of UCP1 demonstrated that HN-001 treatment induced higher expression of UCP1, which was observed in numerous clusters of UCP1-expressing multilocular adipocytes (Figure 6J), suggesting that HN-001 induced thermogenesis in eWAT and sWAT.

**FIGURE 7:**
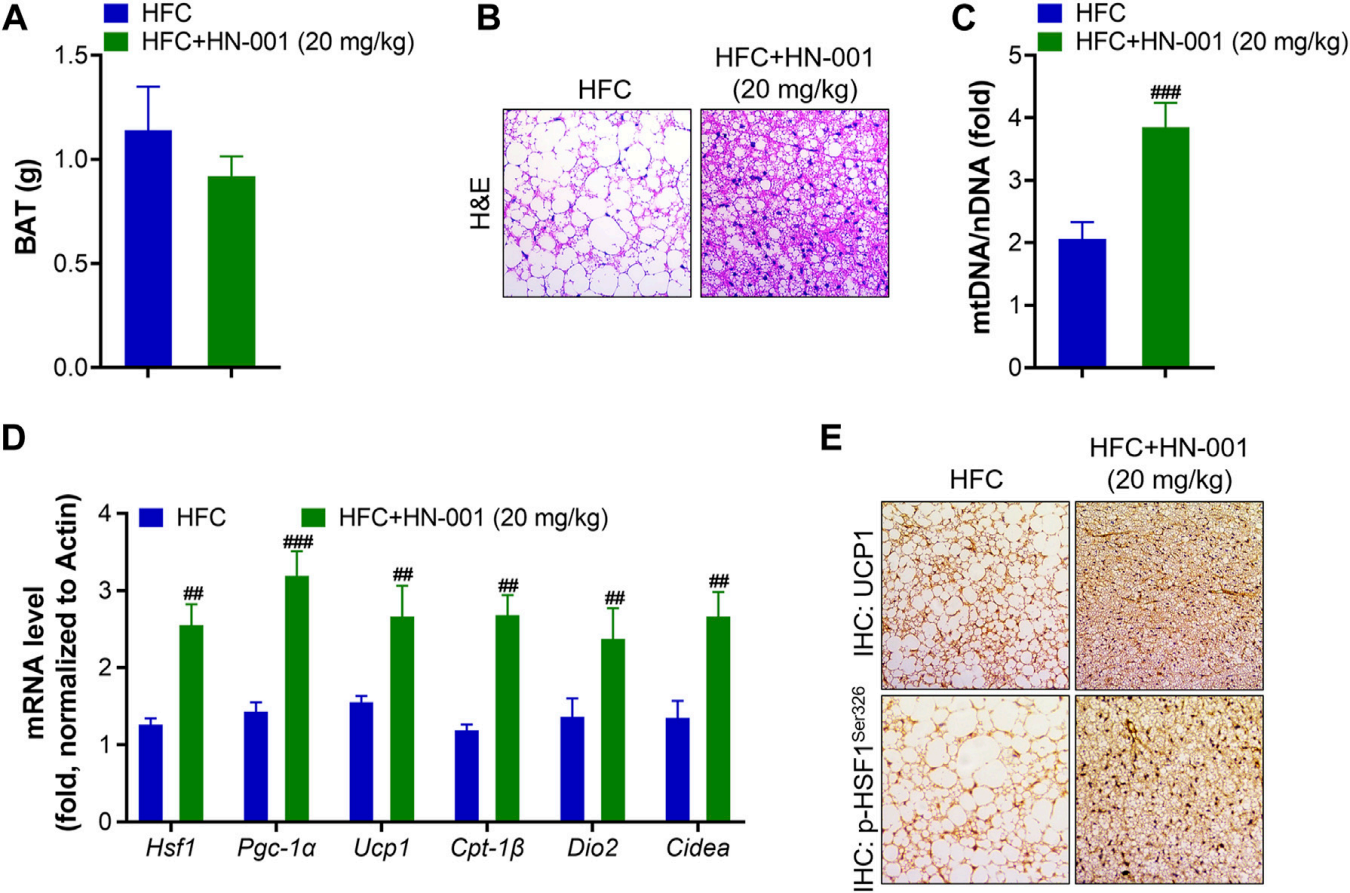
HN-001 enhances thermogenic ability in the BAT of mice. **(A)** BAT mass measurement. **(B)** H&E staining of BAT. Scale bar, 200 μm. **(C)** Mitochondrial content. **(D)** mRNA levels of metabolic and browning regulatory genes. **(E)** Immunohistochemistry analysis of UCP1 and p-HSF1^Ser326^. Scale bar, 200 μm. N = 10 mice/group. ^#^ *p* < 0.05, ^##^ *p* < 0.01, ^###^ *p* < 0.001, compared with HFC control mice.

Consistent with the improved thermogenesis ability, mice treated with HN-001 decreased BAT weight and displayed smaller brown adipocytes, in which the size of lipid droplets was reduced and fewer lipids accumulated in brown adipocytes (Figures 7A, B). This was associated with the increased mitochondrial copy numbers implied by the quantification of mitochondrial DNA (mtDNA) copies (Figure 7C). The increased mitochondrial copy numbers were associated with activation of the expression levels of the HSF1/PGC-1α axis (Figure 7D). Meanwhile, the expression of *Ucp1* and other classical BAT marker genes (deiodinase iodothyronine Type II (*Dio2*) and *cell death inducing DFFA like effector A (Cidea)*) was strongly induced. In the BAT of HN-001-treated obese mice, the expression of mitochondrial biogenesis regulators (*Pgc-1a*) and fatty acid oxidation (*Cpt-1β*) were strongly activated. Immunohistochemistry analysis of p-HSF1^Ser326^ and UCP1 in BAT revealed that HN-001 induced thermogenesis in the BAT of mice (Figure 7E). This evidence suggests that HN-001-activated thermogenic capacity in BAT is closely related to HSF1/PGC-1α axis activation.

## Discussion

The identification of natural therapeutic agents is an attractive strategy for developing new drugs that are more applicable to metabolic disease, as advantages can be taken from the existing knowledge about the safety and molecular mode of actions. Activation of the thermogenic program *in vivo* efficiently enhances energy expenditure and presents a promising avenue for the treatment of obesity (Zhang et al., 2017; Xiang et al., 2018). We recently identified a novel natural HSF1/PGC-1α axis activator isolated from the marine fungus *Aspergillus* c1. sp. using our previously reported high-throughput HSF1/PGC-1α axis activator screening system. HN-001 treatment activated HSF1 and enhanced its transcriptional regulation by enriching the HSF1 protein in the promoter region of DNA *Pgc-1α*, leading to improvements in mitochondrial biogenesis and adaptive oxidation, thus inhibiting adipocyte maturation and lipid accumulation. In obese mice, HN-001 was able to reduce obesity and the accompanying metabolic disorders without detectable tissue breakdown at pharmacological dosages (Rao et al., 2023). These beneficial effects were associated with enhancements of thermogenic ability and activation of the HSF1/PGC-1α axis in adipose tissues. Our findings highlight the importance of activating the HSF1/PGC-1α axis for obesity treatment by increasing adipose thermogenesis.

Restricting energy intake including appetite inhibition and fat absorption and/or promoting energy expenditure is the recommended ways to combat obesity. The induction of a thermogenic program in adipose tissue to promote lipid oxidation is an attractive and promising avenue (Rodrigues et al., 2019). PGC-1α is a master regulator in controlling mitochondrial function and energy metabolism in multiple metabolic organs (Cheng et al., 2018; Kärkkäinen et al., 2019; Buccoliero et al., 2021; Lei et al., 2022; Quattrocelli et al., 2022). In skeletal muscle, PGC-1α is a key regulator of mitochondrial oxidative metabolism and muscle fiber specification (Zhang et al., 2017). In WAT, PGC-1α was rarely expressed and gradually decreased during adipose expansion (Boström et al., 2012). Obese humans have reduced PGC-1α levels in adipose tissue (Yang et al., 2003). In contrast, PGC-1α shows the highest expression in BAT and has some of its most well-established roles. Initially, discovered for its ability to induce mitochondrial biogenesis and adaptive thermogenesis. PGC-1α activation can be achieved through several mechanisms, including gene expression, protein stabilization, and posttranslational modifications. On a transcriptional scale, enriching PGC-1α levels by genetic manipulation (Kleiner et al., 2012) or pharmacological intervention (Qiu et al., 2018; Evans et al., 2019) increased mitochondrial respiration and thermogenic ability in mice. For example, Chen et al. identified a natural DLAT inhibitor, hyperforin (Chen et al., 2021), which activated the AMPK-PGC-1α-UCP1 axis to counteract obesity. PGC-1α has a short protein half-life, and its levels are tightly controlled by several E3 ubiquitin-ligases, making protein stabilization a limiting step in the activation process of PGC-1α and downstream target gene transcription. Recently, Pettersson-Klein et al. (2018) established a cell-based high-throughput PGC-1α protein stabilizer screening system and identified a candidate from 7040 compounds that induced UCP1 expression and enhanced cellular respiration. Given the beneficial metabolic effects of PGC-1α, increased PGC-1α levels in adipose tissues would be an interesting target in the treatment of obesity. Recently, we found that treatment with HN-001 in mice marginally affected energy intake but decreased energy storage efficacy, indicating that HN-001 ameliorated obesity in mice and may correlate with increasing energy consumption. By analyzing body temperature and performing cold tolerance tests, we observed that HN-001 enhanced thermogenesis in mice. This observation was further confirmed by examination of thermogenic and metabolic-related markers in adipose tissues, including BAT and WAT. Consistent with these findings, treatment of adipocytes or mice with HN-001 increased PGC-1α levels at both the transcriptional and translational scales. These data coincided well with the beneficial role of PGC-1α in adipose tissues.

HSF1 is a classical transcription regulator that can be activated upon heat shock or metabolic insults. Our and other studies have uncovered a critical role of HSF1 as a regulator of brown fat and mitochondrial oxidation through regulating the expression of browning markers, including PGC-1α and A2b1 (Li et al., 2022a; Li et al., 2022b). Previously, we established a novel HSF1/PGC-1α axis activator screening system and identified the clinical hepatoprotective agent matrine as a robust HSF1/PGC-1α axis activator that efficiently alleviated obesity by inducing a thermogenic program in mice (Li et al., 2022a). This study verified a proof of principle that pharmacological activation of the HSF1/PGC-1α axis effectively counteracts obesity and induces a thermogenic program in adipose tissue. By using the screening system, we recently identified a novel natural compound HN-001, as a potential HSF1/PGC-1α axis activator, which was reported to ameliorate MAFLD in mice by us Rao et al. (2023). Treatment of adipocytes or mice with HN-001 efficiently activated HSF1 and enriched it in the promoter region of *Pgc-1α*, increasing PGC-1α expression levels. Deletion of HSF1 decreased *Pgc-1α* levels and abolished the stimulation effect of HN-001, including thermogenic ability, mitogenesis, mitochondrial oxidation, lipid-decreasing ability and the expression level of PGC-1α, further confirming the effect of HSF1 in regulating PGC-1α expression.

**FIGURE 8:**
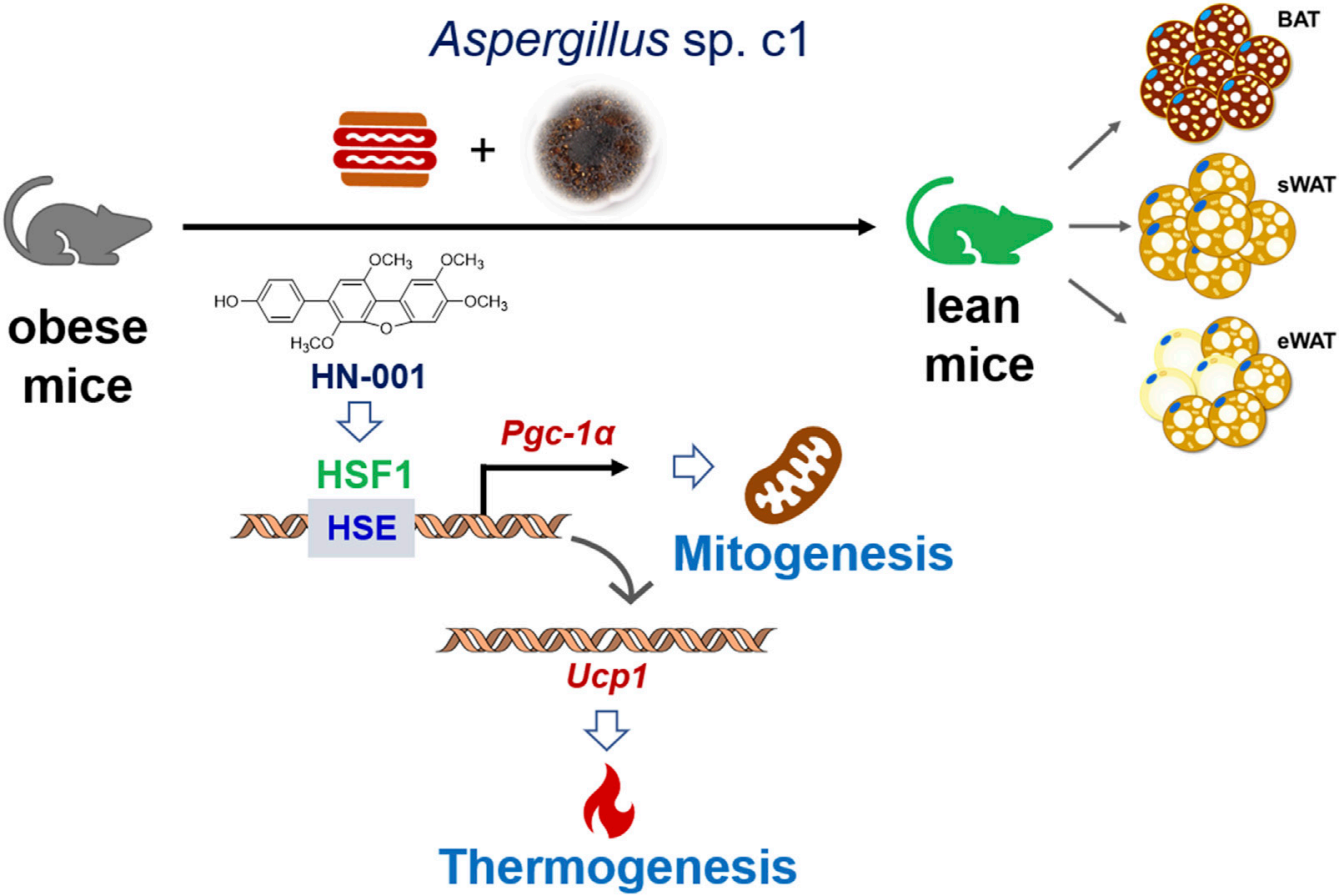
Induction of the thermogenic program in adipose tissue in HN-001-treated DIO mice. As exemplified by HN-001, activation of the HSF1/PGC-1α axis by enriching the phosphorylated HSF1 to the promoter of PGC-1α then activated the expression of UCP1, leading to mitogenesis and thermogenesis in adipose tissue, thus inhibiting adipocyte maturation. As a result, obesity is alleviated in mice. Abbreviations: BAT, brown adipose tissue; sWAT, subcutaneous white adipose tissue; eWAT, subcutaneous white adipose tissue; HSE, heat shock element; HSF1, heat shock Factor 1; PGC-1α, PPARγ coactivator 1α. UCP1, uncoupling protein 1.

Overall, our study identified a natural HSF1/PGC-1α axis activator from the marine fungus *Aspergillus* c1. sp. Treating mice with HN-001 efficiently attenuated obesity by inducing a thermogenic program alongside HSF1/PGC-1α axis activation in adipose tissue (Figure 8/Graphic abstract), indicating that HN-001 is an interesting candidate for the treatment of metabolic disorders.

## Data Availability

The raw data supporting the conclusion of this article will be made available by the authors, without undue reservation.

## Abbreviations

ACC, acetyl-CoA carboxylase; BAT, brown adipose tissue; CIDE, A, cell death inducing DFFA like effector A; CPT-1β, carnitine acyl transferase 1β; DIO2, iodothyronine deiodinase Ⅱ; FASN, fatty acid synthase; GTT, glucose tolerance test; HFC, high-fat and high-cholesterol diet; HSE, heat shock element; HSF1, heat shock factor 1; ITT, insulin tolerance test; mtDNA, mitochondrial DNA; NEFA, Non-esterification fatty acid; NRF2, nuclear factor E2-related factor 2; PGC-1α, peroxisome proliferator-activated receptor-γ coactivator-1α; SREBP-1c, sterol regulatory element binding protein-1c; TFAM, transcription Factor A, mitochondrial; TG, triglyceride; UCP1, uncoupling protein 1; WAT, white adipose tissue.
