# Supplementary material for: Marine fungus Aspergillus c1. sp metabolite activates the HSF1/PGC-1α axis, inducing a thermogenic program for treating obesity
Source: Front Pharmacol. 2024 Jan 25;15:1320040. doi: 10.3389/fphar.2024.1320040 (PMC10851286; doi:10.3389/fphar.2024.1320040)
Supplement: Supplementary file 1 [file DataSheet1.PDF]

Supporting information

for

Marine fungus *Aspergillus* c1. sp metabolite activates the HSF1/PGC-1α axis, inducing a thermogenic program for treating obesity

Yong Rao*[, †], Rui Su[†], Chenyan Wu, Guanyu Yang, Renquan Fu, Junjie Wu, Jinqiang Liang, Jin Liu, Zhongping Jiang, Congjun Xu, Ling Huang*

[a] *Key Laboratory of Tropical Biological Resources of Ministry of Education, School of Pharmaceutical Sciences, Hainan University, Haikou 570200, China*

* **Correspondence**
Professor Yong Rao and Ling Huang, School of Pharmaceutical Sciences, Key Laboratory of Tropical Biological Resources of Ministry of Education, Hainan University, Haikou 570200, China.
E-mail addresses: raoyong@hainanu.edu.cn (Yong Rao); Linghuang@hainanu.edu.cn (Ling Huang).
Tel./Fax: +86-13724891401; +86-13631339351;
[†]These authors made equal contributions to this work.

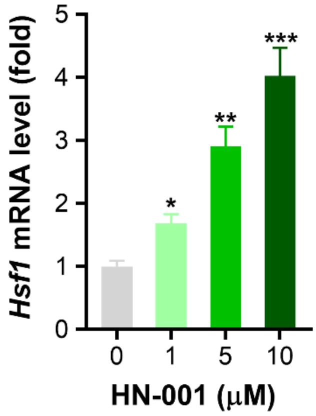

Figure S1. Examination of effect of HN-001 on the transcription level of Hsf1 in adipocytes. C3H10-T1/2 derived adipocytes treated with vehicle or HN-001 for 24 h, cells were harvested for *Hsf1* mRNA analysis. N = 3 independent biological experiments. * $p < 0.05$, ** $p < 0.01$, *** $p < 0.001$, compared with control cells.

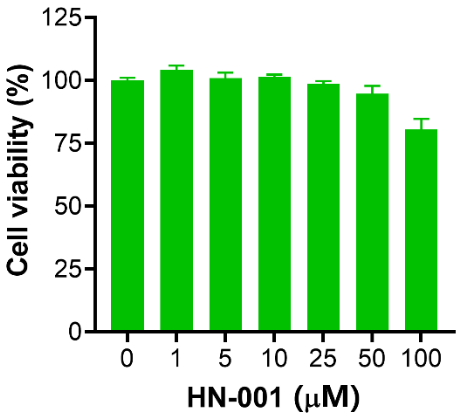

Figure S2. Examination of cytotoxicity of HN-001 against C3H10-T1/2 cells. C3H10-T1/2 cells were treated with vehicle or HN-001 for 24 h, cells were harvested for cell viability analysis. N = 3 independent biological experiments. * $p < 0.05$, ** $p < 0.01$, *** $p < 0.001$, compared with control cells.
